# Polyacrylamide Hydrogel Containing Calendula Extract as a Wound Healing Bandage: In Vivo Test

**DOI:** 10.3390/ijms24043806

**Published:** 2023-02-14

**Authors:** Lindalva Maria de Meneses Costa Ferreira, Elanne de Sousa Bandeira, Maurício Ferreira Gomes, Desireé Gyles Lynch, Gilmara Nazareth Tavares Bastos, José Otávio Carréra Silva-Júnior, Roseane Maria Ribeiro-Costa

**Affiliations:** 1 Laboratory of Nanotechnology Pharmaceutical, Institute of Health Sciences, Federal University of Pará, Rua Augusto Corrêa 01, Belém 66075-110, PA, Brazil; 2 Laboratory of Neuroinflammation, Institute of Biological Sciences, Federal University of Pará, Augusto Corrêa 01, Belém 66075-110, PA, Brazil; 3 School of Pharmacy, College of Health Sciences, University of Technology, 237 Old Hope Road, Kinston 6, Jamaica; 4 Laboratory R&D Pharmaceutical and Cosmetic, Institute of Health Sciences, Federal University of Pará, Rua Augusto Corrêa 01, Belém 66075-110, PA, Brazil

**Keywords:** hydrogel, polyacrylamide, wound healing, dressing and calendula extract

## Abstract

Hydrogel is a biomaterial widely used in several areas of industry due to its great biocompatibility and adaptability to biological tissues. In Brazil, the Calendula plant is approved by the Ministry of Health as a medicinal herb. It was chosen to be incorporated in the hydrogel formulation because of its anti-inflammatory, antiseptic and healing effects. This study synthesized polyacrylamide hydrogel containing calendula extract and evaluated its efficiency as a bandage for wound healing. The hydrogels were prepared using free radical polymerization and characterized by Scanning Electron Microscopy, swelling analysis and mechanical properties by texturometer. The morphology of the matrices showed large pores and foliaceous structure. In vivo testing, as well as the evaluation of acute dermal toxicity, was conducted using male Wistar rats. The tests indicated efficient collagen fiber production, improved skin repair and no signs of dermal toxicity. Thus, the hydrogel presents compatible properties for the controlled release of calendula extract used as a bandage to promote cicatrization.

## 1. Introduction

Skin injuries caused by trauma and other accidents often lead to a range of health complications and sometimes death [1,2]. Wound healing for skin wounds is a physiological process that depends on molecular and cellular mechanisms. The process is divided into three phases: the inflammatory, proliferative and remodeling phase [3,4,5,6]. Acceleration of healing requires the maintenance of wound sterility, pain reduction, elimination of exudate, possibility of gas exchange, ease of handling and reduction in the number of dressing applications [7,8]. Although there are several treatment options on the market for the treatment of skin wounds, most tend to be expensive for the patient, due to the long-term treatment required [8,9]. Ideal dressings have the following criteria: providing and maintaining a moist environment, protecting the wound against secondary infections, allowing gas exchange, providing thermal insulation, being free of toxic particles or contaminants, handling excess exudate, being elastic, having a low cost, durability, flexibility and mechanical strength [8,10,11]. The main advantage of modern bandages is their ability to retain and build a humid ambient around the injury in order to encourage the cicatrization process [6,10,12]. Several skin bandages have been designed using advanced technologies, and are regarded as suitable options, including: fiber bandage wraps, gauze, medical film and hydrogels [9,10,13,14,15]. 

Hydrogels are three-dimensionally reticulated polymeric matrices that have the ability to absorb large amounts of water and biological liquids [16,17,18,19]. Due to their great capacity to absorb water, the presence of cavities and their smooth firmness, they mimic natural living tissues more than any other class of biomaterials [20,21]. They are widely used in the treatment of wounds, promoting autolytic debridement, thus maintaining a moist environment around the wound, which accelerates cicatrization [22,23,24]. The properties of these biomaterials, such as their biocompatibility, high oxygen permeability, wound moisture retention and absorbability, all help to increase patient compliance [22,25]. The smooth nature of hydrogels allow them to be easily removed from the skin without causing any irritation or additional damage [22,26,27]. However, the practical applications of hydrogels as bandages are still currently limited, due to their poor mechanical strength and stability [3,28]. The development of a new hydrogel with superior properties becomes necessary for application in the skin wound healing process. The use of hydrogels as dressings for wound treatment applications has been reported in several studies [29,30]. 

Among the polymers utilized in hydrogel formulations, polyacrylamide (synthetic) and methylcellulose (natural) stand out [29,31]. Polyacrylamide (PAAm) has low toxicity and is economical. It is synthesized using free radical polymerization in aqueous solution, or solid state crystalline acrylamide polymerization using ionizing radiation [32,33]. The main characteristic of the PAAm hydrogel is its degree of swelling, which gives great flexibility and cohesion, and which provides a granulation and epithelializing effect which is an ideal property for a dressing [23,34]. Methylcellulose (MC) originates from the methylation process of the natural polymer cellulose. When MC is cross-linked in the PAAm network with irreversible covalent bonds, a resistant biomaterial is created, which has the great advantage of being simple and cheap, thus favoring its economic viability [35,36]. The combination of the two polymers with different characteristics result in the formation of a new polymer with advanced mechanical strength, biocompatibility and biodegradability [3,37,38].

Ideal dressings also need to exhibit anti-inflammatory, healing, antioxidant and antibacterial activity [39,40]. *Calendula officinalis* L. or calendula, belongs to the *Asteraceae* family. It is native to southern Europe and was used not only for decorative purposes but also as a medicinal herb [41,42,43,44]. Among the bioactive compounds found in the species, it contains carotenoids, lycopene, phenolic acids, hydroxycinnamic acids (p-coumaric, caffeic, chlorogenic acids), flavonoids (rutin) and coumarins (esculetin) [44,45].

The presence of phenols in the species contributes to its high antioxidant potential [46,47]. Calendula has widespread therapeutic applications, which include tissue re-epithelialization and general wound healing action. [43,48]. Thus, this study is aimed at developing a polyacrylamide-based hydrogel containing calendula extract to be used as a dressing for wound healing. 

## 2. Results and Discussion

### 2.1. Preparation of Hydrogel

**Figure 1:**
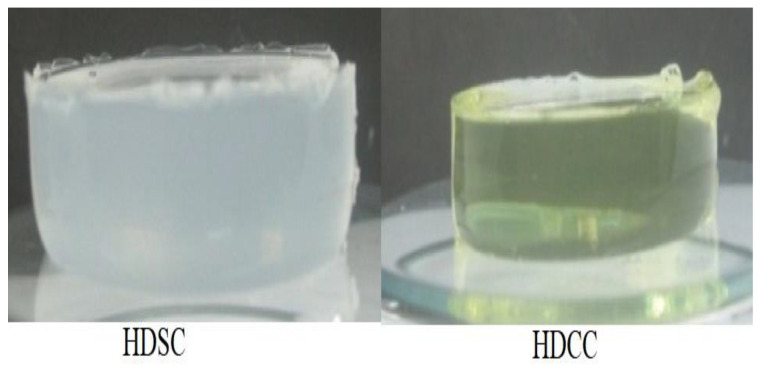
Appearance of white hydrogel (**left**) and hydrogel containing calendula extract (10%) after the free radical polymerization reaction (**right**).

White hydrogels and the hydrogel containing the calendula extract had a gelatinous and translucent appearance. However, the hydrogel containing the extract had a yellow hue, obtained from the calendula extract (Figure 1). The white hydrogel showed to be visibly resistant and with intensified color. 

### 2.2. Characterization

#### 2.2.1. Morphology Analysis

**Figure 2:**
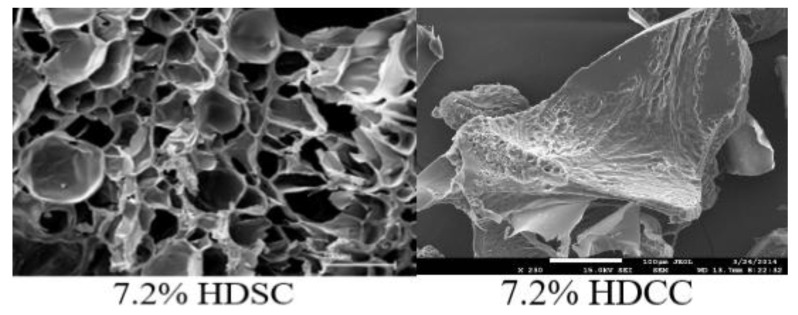
SEM micrographs of 7.2% HDSC (**left**) and 7.2% HDCC (**right**) in magnification 1400×.

Scanning Electron Microscopy (SEM) micrographs of the 7.2% HDSC (without calendula extract) and 7.2% HDCC (with calendula extract) are shown in Figure 2. The photomicrographs of the matrices without the addition of the extract showed clear pores and foliaceous structure, characteristic of the three-dimensional network the hydrogels [23]. The 7.2% HDSC showed large pores, having irregular and non-uniform shapes (Figure 2). The porous structure has the potential to absorb exudate exiting the wound, in addition to helping to diffuse nutrients and healing promoters to the site, while still maintaining an appropriate moist environment [9].

The photomicrographs of the matrices containing the calendula extract showed that there was a filling of all pores in the polymer matrix. The addition of the extract altered the shape and structure of the pores. The structure of hydrogels 7.2% acrylamide found in this research was observed in a previous study by Gyles at al. [23]. Where the morphological characteristics shown in the hydrogel matrix containing calendula extract were similar to the study which showed the incorporation of *Aloe barbadensis* extract [23], and another study which showed the incorporation of *Aloe arborescens* aqueous extract within the hydrogel matrix [49], the present study suggests that the hydrogel matrix can be compatible for the incorporation of plant extracts.

#### 2.2.2. Swelling Studies

**Figure 3:**
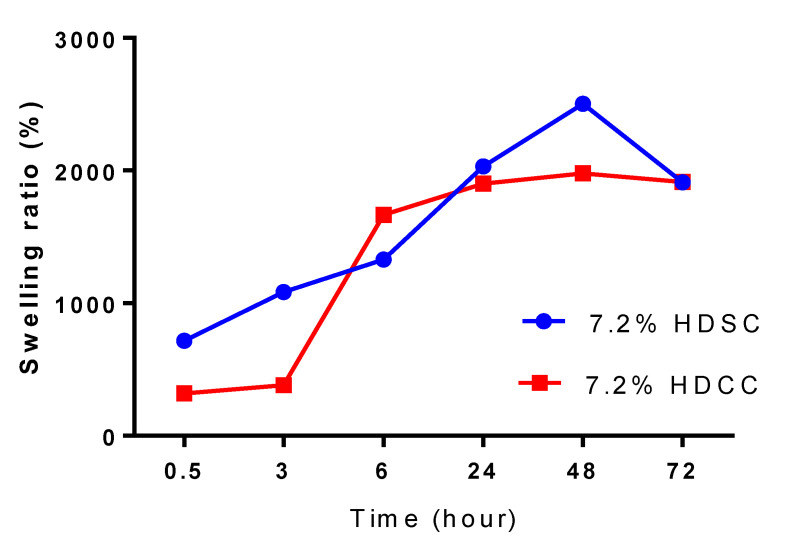
Degree of swelling of 7.2% HDSC and 7.2% HDCC for a period of 72 h.

The hydrogels 7.2% HDSC and 7.2% HDCC were evaluated for the degree of swelling, the results are shown in Figure 3. In this study, the values for 7.2% HDSC ranged between 715% and 2500% while the 7.2% HDCC values ranged from 318% to 1979%. The swelling behavior of a hydrogel is one of the most essential characteristics for bandages as it provides a moist environment in the wound area, in addition to being directly related to the ability to absorb wound exudate, prevent infections and facilitate the healing process [50,51].

Polyacrylamide absorbs water through the formation of hydrogen bonds using osmosis as the mechanism of action. In addition, methylcellulose has hydroxyl groups, which also account for greater water absorption by the hydrogel [23]. The experiment lasted a period of 72 h, and its swelling profile was not constant, which consequently could lead to an even greater water absorption. The porous structure of the hydrogel may also have influenced an increase in water absorption. There was a significant increase in the hydrogel size during the swelling process, the polymer matrix became more flexible and absorbed a large amount of water at room temperature. The hydrogels with and without the calendula extract showed a great capacity for water absorption, HDSC swelled to 2500% and HDCC to 1979% in 48 h.

The incorporation of the extract in the polymer matrix did not change the profile of the hydrophilic property of the polyacrylamide hydrogels. The water absorption behavior found in this study is similar to previous studies by Gyles et al. [23], Xue et al. [24] and Zakerikhoob et al. [10]. The polyacrylamide hydrogels, due to their great water absorbing potential, are characterized as an efficient biomaterial when applied directly to the wound for hydration.

#### 2.2.3. Mechanical Properties by Texturometer

**Figure 4:**
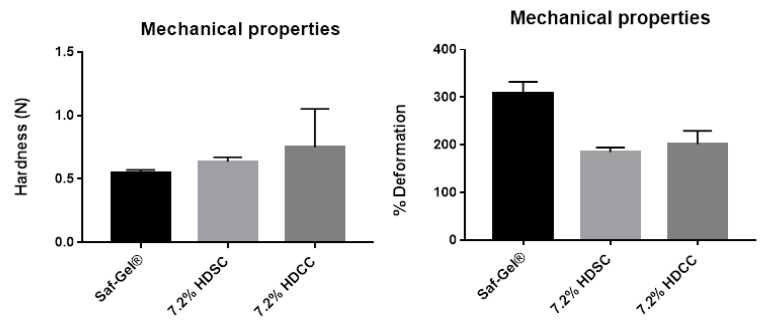
Mechanical test of 7.2% HDSC, 7.2% HDCC and control Sal-Gel^®^ in texturometer.

Mechanical resistance is a very important property in hydrogels that are used as bandages for wound treatment. Good mechanical strength maintains its integrity when skin tissue is damaged by external forces and protects the wound [52]. The mechanical properties of the hydrogels were evaluated in terms of tensile strength and deformation rate (Figure 4). The 7.2% HDSC showed a tensile strength value of 0.64 ± 0.04 N and deformation 185.5 ± 9.19%. The 7.2% HDCC showed a tensile strength value of 0.75 ± 0.36 N and deformation 201.7 ± 28.22%. The commercial product used as a control showed a tensile strength value of 0.55 ± 0.03 N and deformation 307 ± 25.3%. Mechanical strength values of 1.447 ± 0.108 N (pH 2.2 buffer), 0.786 ± 0.081 N(DW), 0.779 ± 0.117 N(PBS) and 0.553 ± 0.061 N (SWF) in hydrogel films were reported by Singh et al. [53]. The 7.2% HDSC and 7.2% HDCC showed low mechanical resistance and high deformation rate; however, their values were higher than the commercially sold product used in the study as a control.

The preliminary test of the mechanical property of hydrogels did not show a positive result as expected, with a concentration of 7.2% polyacrylamide. The mechanical strength of hydrogels can be improved with the addition of reinforcing agents, subsequent complexation of ions and strong covalent crosslinking during synthesis methods [54]. Although the result was not what was expected, it leaves room for optimizing the formulation, perhaps with an increase in the concentration of the polymer that presents this property of improving the resistance of hydrogels or the insertion of a second polymer that has this characteristic, such as alginate of sodium.

### 2.3. Hydrogel Biocompatibility Study

#### 2.3.1. Acute Dermal Toxicity Test

**Figure 5:**
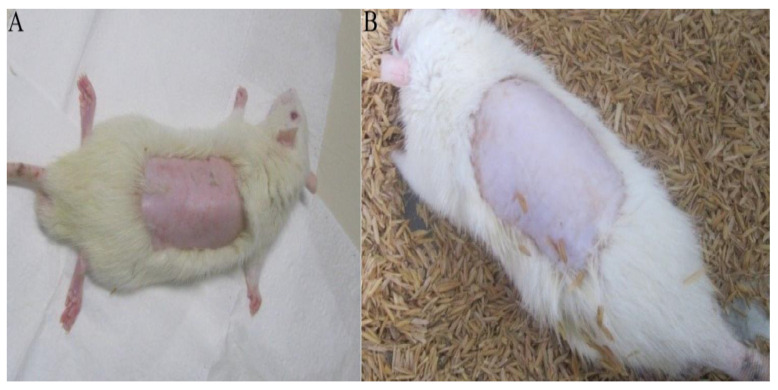
Images showing animal epilation prior to application of hydrogel for dermal toxicity test (**A**) and animal 24 h after hydrogel removal in acute toxicity test (**B**).

Acute toxicity tests are preliminary in evaluating the safety of a product and predicting possible adverse effects. In this study, there was no evidence of changes in the specimens observed. The animals maintained constant weight, the skin showed no signs of toxicity, and no systemic alteration, indicating toxicity, was observed. The food/water ratio remained the same, without any changes in behavior of animals. After 24 h hydrogel was removed from the animals and no macroscopic change was observed (Figure 5).

Cascone and Lamberti [29] observed that the use of polyacrylamide in the hydrogel synthesis did not present a risk, as it is a commonly used biomaterial in the pharmaceutical industry. Methylcellulose, a natural and biocompatible polymer is also included in its composition, making up a part of the three-dimensional structure. The skin to which the hydrogel was applied showed a better appearance, indicative of better tissue hydration. Water retention is an essential property for a wound bandage, as it keeps the wound hydrated and facilitates exchanges, in addition to promoting the healing process (Figure 5) [20,51].

#### 2.3.2. Evaluation of Hydrogel Action in Healing Process

**Figure 6:**
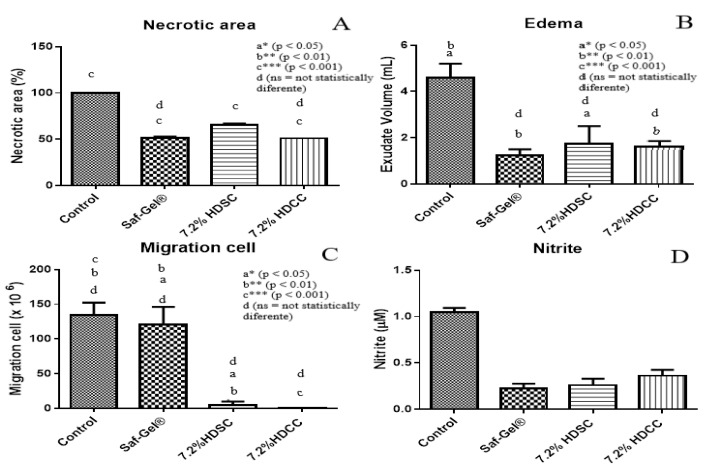
The effect of different samples of wound healing in ischemia-reperfusion model. (**A**) Necrotic area, (**B**) Edema, (**C**) Migration cell and (**D**) Nitrite. The *p*-value was compared between groups was admitted to be statistically significant when *p* < 0.05. Different letters in the same column indicate significant difference: a * (*p* < 0.05), b ** (*p* < 0.01), c *** (*p* < 0.001), d (ns = not statistically different).

The efficacy of hydrogel as a wound healing agent was investigated in vivo (Figure 6). Analysis was performed using the ischemia-reperfusion model to create a pressure wound. The rats were randomly divided into groups, where they were treated using the following: saline solution (negative control), SAF-gel^®^ (positive control), 7.2% HDSC and 7.2% HDCC. Tukey’s test was used to compare group results. The analysis was conducted by measuring the initial necrotic area and taking it away from the necrotic area after treatment. The results showed a significant reduction in wound circumference, as a result of wound contraction in the sample treated groups. The tested hydrogels presented positive effects on the tissue regeneration process, as observed in the reduction in wound size in the treated groups. The 7.2% HDCC showed the most significant decrease in wound size which was equivalent to the SAF-gel^®^ positive control with around 50% reduction in the total ulcer size (Figure 6). It is believed that hydrogels directly contribute in the tissue repair process. The edema extravasation was measured by the volume of exudate formed at the wound site. The treated groups with 7.2% HDCC and 7.2% HDSC group presented a significant decrease in volume of exudate produced, in comparison to the negative control group. 7.2% HDCC and HDSC did not show any significant difference to the positive control SAF-gel^®^, which was probably due to the high concentration of polyacrylamide, known to have positive effects on wound granulation and epithelialization (Figure 6A). 

In Tukey’s multiple comparison test, negative control and SAF-gel^®^ showed a statistically significant difference (*p* < 0.001). Negative control and 7.2% HDSC showed a statistically significant difference (*p* < 0.001). Negative control and 7.2% HDCC showed a statistically significant difference (*p* < 0.001). SAF-gel^®^ and 7.2% HDSC showed a statistically significant difference (*p* < 0.001). SAF-gel^®^ and 7.2% HDCC were not statistically different. 7.2% HDSC and 7.2% HDCC showed a statistically significant difference (*p* < 0.001) (Figure 6A).

Hydrogels are designed for the facilitation of the debridement and hydration of necrotic tissues or devitalized areas, in addition to facilitating exudate removal, stimulating tissue granulation, epithelialization and the filling of cavities. The reduction in the volume of exudate demonstrated a possible anti-inflammatory activity of the hydrogels and that result could be directly linked to induced cellular edema. The 7.2% HDCC and HDSC can be used as dressings for the treatment of tissue injuries, such as pressure injuries (Figure 6B) [55,56]. 

In Tukey’s multiple comparison test, negative control and SAF-gel^®^ showed a statistically significant difference (*p* < 0.01). Negative control and 7.2% HDSC showed a statistically significant difference (*p* < 0.05). Negative control and 7.2% HDCC showed a statistically significant difference (*p* < 0.01). SAF-gel^®^ and 7.2% HDSC were not statistically different. SAF-gel^®^ and 7.2% HDCC were not statistically different. 7.2% HDSC and 7.2% HDCC were not statistically different (Figure 6B).

In assessing the cell migration, the reduction in the number of cells in the lesion site indicates the anti-inflammatory potential. An amount of 7.2% HDCC and HDSC decreased the total number of cells compared to the other groups presented (Figure 6C), this indicates that hydrogels have an effect on decreasing the cell migration process, a fact that corroborates their anti-inflammatory action. The macrophage is the most important inflammatory cell in this phase and is maintained from the third to the tenth day, along with phagocytes bacteria, debrided foreign bodies and the development of granulation tissue. High phagocytic activity of macrophages was observed after trauma [6]. 

In Tukey’s multiple comparison test, negative control and SAF-gel^®^ were not statistically different. Negative control and 7.2% HDSC showed a statistically significant difference (*p* < 0.01). Negative control and 7.2% HDCC showed a statistically significant difference (*p* < 0.001). SAF-gel^®^ and 7.2% HDSC showed a statistically significant difference (*p* < 0.05). SAF-gel^®^ and 7.2% HDCC showed a statistically significant difference (*p* < 0.01). 7.2% HDSC and 7.2% HDCC were not statistically different (Figure 6C).

In the evaluation of nitric oxide (NO) activity, there was no interference of NO production in the different formulations, thus showing that calendula has no activity against NO production (Figure 6D).

#### 2.3.3. Histopathological Evaluation

**Figure 7:**
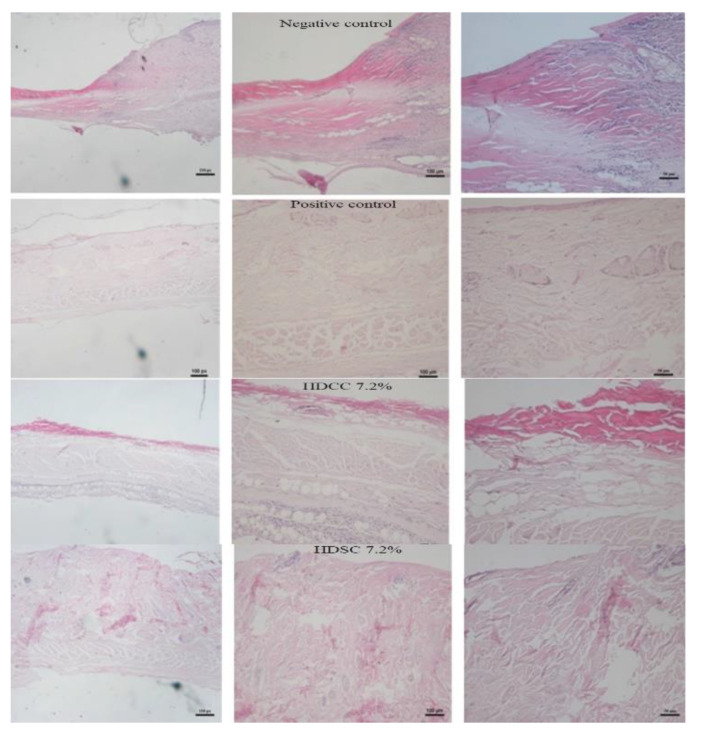
Histological analysis by hematoxylin and eosin presenting the characteristics tissue structures and cellular infiltrates of negative control, positive control, 7.2% HDSC 7.2% and 7.2% HDCC in evaluation of wound healing.

The histological analysis of wound healing, which indicates the structural or qualitative characteristics of tissues and cellular infiltrates, was performed. The negative control group, treated only with saline solution, showed a depletion of the skin layers, representing the invasiveness of the lesion. The positive control (SAF-gel^®^) demonstrated a reduction in the rate of cellular infiltrates and an organization of tissue layers. The group treated with 7.2% HDSC and the group treated with 7.2% HDCC (Figure 7) had low levels of cellular infiltrates and good tissue organization, which was similar to that of the positive control.

The sections stained with HDCC 7.2% and the HDSC 7.2% group had similar results to the SAF-gel^®^, which showed tissue organization and a significant reduction in cellular infiltrates (mainly macrophages and leukocytes) to the injured tissues.

**Figure 8:**
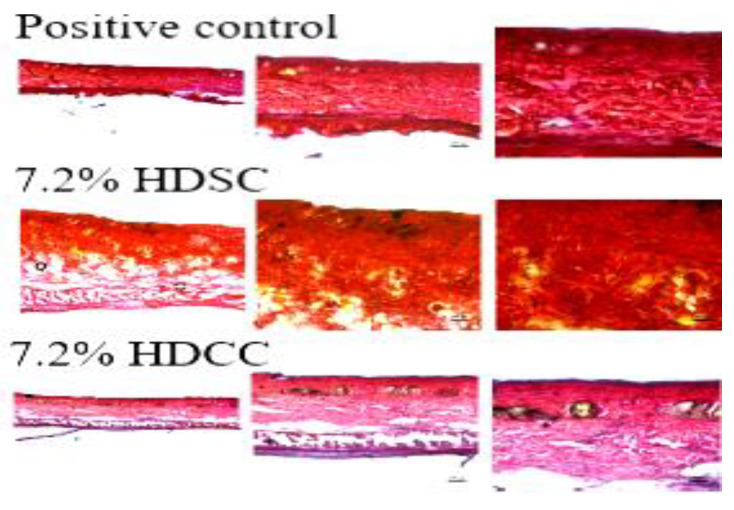
The histopathological images for the effect of positive control, 7.2% HDSC 7.2% and HDCC of wound healing using the Picrosirius–Hematoxylin.

The evaluation of collagen by skin staining was conducted using the Picrosirius–Hematoxylin technique. The collagen fibers were stained, and the different types were analyzed for organization of tissue restoration (Figure 8). 

The 7.2% HDCC and 7.2% HDSC (Figure 8) showed a significantly red contrast represented by the collagen fibers present, this result was similar to results of the positive control. Treatment with 7.2% HDCC and 7.2% HDSC appeared to contribute directly to the tissue restoration process, highlighting the possible anti-inflammatory and healing potential, confirming its use in future applications as a dressing for tissue injuries.

This research showed that calendula extract, associated with the polyacrylamide hydrogel matrix, can be effective in the treatment of skin wounds, because it provides perfect conditions for faster, more effective wound healing. The bandage made from the formulation also showed a reduction in the amount of exudate as well as macrophage proliferation. Afrin et al. [57] and Zhang et al. [58] also confirmed the efficiency of hydrogels in the wound healing process in an in vivo study. The anti-inflammatory action observed in the results was attributed to flavonols (specifically rutin) present in the calendula extract. This anti-inflammatory activity is scientifically proven and has been shown to be efficient for healing skin wounds, making it ideal to be used as a bandage. This study confirms the importance of the hydrogel matrix as a release mechanism [59], for drug substances, extracts [60] and liposomes loaded with resveratrol [61]. There is a possible application of this release mechanism in the pharmaceutical and cosmetic areas. Given the positive results obtained, future studies will involve performance tests to characterize the hydrogel, in addition to studies that prove the mechanism of action of the flavonoids responsible for promoting anti-inflammatory action.

## 3. Materials and Methods

### 3.1. Materials

Acrylamide ≥ 99 % (AAm), Sodium persulfate ≥ 98 % (SP), methylcellulose viscosity: 15 cP (MC), N,N,N,N-tetramethylethylenediamine 99% (TEMED), N,N′-Methylenebisacrylamide 99 % (MBAAm) were purchased from Sigma-Aldrich (St. Louis, MO, USA). All chemicals and solvents of analytical reagent grade were obtained from LabSynth (São Paulo, Brasil). 

### 3.2. Preparation of Calendula Extract

The calendula extract was obtained using the percolation method. One kilogram of the plant drug was placed in the hydrothanic solution at 70% and left to be macerated for 72 h. After this period, the mixture was percolated for 5 days. The extracted solution was concentrated using a rotary evaporator (Buchi R-210, Geneva, Switzerland) at a controlled temperature (40 ± 2 °C) until all the solvent was evaporated. The remaining crude extract (CE) was placed in an amber flask and maintained under refrigeration until the analysis [62].

### 3.3. Synthesis of Hydrogel

White hydrogels (HDSC) and hydrogels with calendula extract (HDCC) were synthesized using free radical polymerization. The synthesis was carried out in aqueous solution, adding the monomer AAm 7.2% *m*/*v*, 0.5% MC *m*/*v*, the crosslinking agent MBAAm (8.55 µmol·mL^−1^). The reaction initiator was then placed in the reaction medium, sodium persulfate (SP) (3.38 µmol·mL^−1^) and the catalyst TEMED (3.21 µmol·mL^−1^). For the synthesis of HDCC, calendula extract was incorporated at a concentration of 100 mg/mL before being added to the reaction medium containing the reaction initiator sodium persulfate and the catalyst. The solution was placed under nitrogen (N_2_) atmosphere for 20 min [23]. The hydrogels were then placed on dialysis for 3 days in distilled water and the water was changed every 24 h. The hydrogels were lyophilized for the in vivo study and analysis of the degree of swelling. In order to carry out the scanning electron microscopy analysis, the hydrogels were micronized (Pulverisette 14 Fristch, Idar-Oberstein, Germany) with a 500 µm mesh.

### 3.4. Characterization of Hydrogels

#### 3.4.1. Surface Morphology Analysis

The morphological analysis of the hydrogels was performed using the Scanning Electron Microscope (LEO-ZEISS, 1450 VP, Jena, Germany). The micronized hydrogels were deposited on a sample holder with the aid of carbon adhesive tape and coated with a layer of gold (Au) 15 nm thick for 1.5 min and observed in secondary electrons and magnification 1400× [23].

#### 3.4.2. Swelling Assay

The 7.2% HDSC and 7.2% HDCC were immersed in deionized water until they reached swelling equilibrium. After blotting off the excess water, their weights were recorded and denoted as Weq. The hydrogels were stored at 37 °C while the tests were being conducted. Their weights were measured and recorded at predetermined time intervals [63]. The swelling behavior of the hydrogels was obtained using the following equation:

(1) $$\mathrm{Swelling}\;\mathrm{behavior}\;(\%)=\frac{\mathrm{Wt}}{\mathrm{Weq}}\times 100\%$$

where Wt and Weq represent the time-dependent and initial (t = 0 min) weight of hydrogels, respectively.

#### 3.4.3. Mechanical Properties by Texturometer

The mechanical properties of 7.2% HDSC, 7.2% HDCC and SAFGel^®^ control were evaluated by tensile test using a texturometer (Brookfield CT-3 Texture Analyzer, Berlin, Germany). The hydrogels were fixed on 4 mm diameter roller grip probes and compressed at a speed of 0.5 mm/s until ruptured. The analysis was performed in triplicate [64,65].

### 3.5. Hydrogel Biocompatibility Study

#### 3.5.1. Animal Test Subjects 

Wistar adult male rats weighing between 200 and 300 g were used in the experiment obtained from the Animal Facility of the Federal University of Pará. The animals were kept in individual cages at temperature (24 ± 3 °C), standard forage, fed with food and water ad libidum and light/dark cycle of 12 h.

#### 3.5.2. Acute Dermal Toxicity Test 

**Figure 9:**
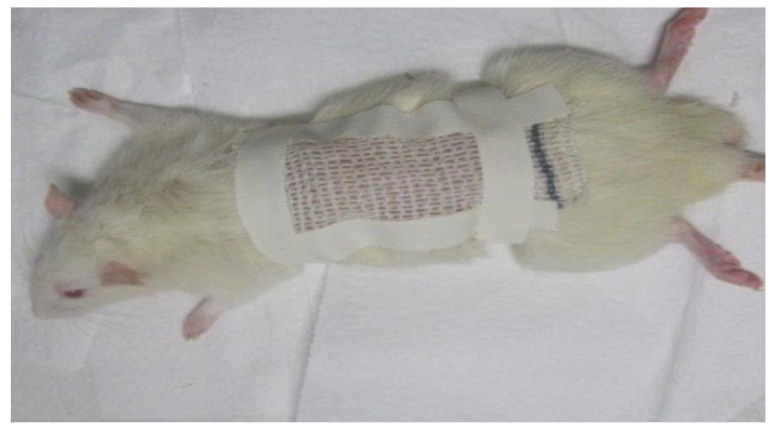
Animal after epilation, in treatment with hydrogel in acute dermal toxicity assay.

Seventeen healthy rats were divided into the 4 groups used for each dose (2 animals being used as control). Twenty-four hours before the experiment, the hairs on the dorsal region of the animals’ trunk were removed by epilation, about 10% of the total body surface area, keeping the animal’s skin without damage (Figure 9). Samples of HDSC and HDCC measuring approximately 5 cm^2^, were applied to the animal’s dermis and kept in contact with the animal’s skin for 24 h. After this period, samples been removed and effects on the dermis were evaluated. The animals were observed for 14 days, then submitted to euthanasia, under the administration of a lethal dose of thiopental [66].

#### 3.5.3. Ulcer Formation

The biocompatibility study of the hydrogels was carried out using the ischemia and reperfusion pressure ulcer model. Thirty-two adult male rats were separated into four groups. All the test rats underwent the cycles of ischemia and reperfusion to form the wound and treated with HDSC, HDCC, negative control (saline solution) and positive control (SAFGel^®^). The wound was created after epilation using a surgical procedure where a sterilized steel plate was inserted into the animal’s dorsal region. The animals were anesthetized with an anesthetic solution of ketamine (20 mg/mL), xylazine (4 mg/mL), acepran (2 mg/L) and diazepam (0.3 mg/mL, diluted in saline solution). Volume of 200 µL was administered for every 100 g of animal. Twenty-four hours after insertion of the plate, 4 cycles of ischemia and reperfusion were performed per day, consisting of 2 h of ischemia and 30 min of reperfusion. The pressure was applied using a 2 × 1 × 1 cm magnet of 1250 Gauss [67]. The treatment lasted for three days, after which the animals were submitted to euthanasia, under the administration of a lethal dose of thiopental [66]. 

##### Exudate Formation Assessment

The exudate was collected through an incision at the wound site and removal of the steel plate. After that, 1 mL of saline solution was placed in the place where the plate was and later, with the aid of an automatic pipettor, the volume total was withdrawn and accounted for [67].

##### Cell Migration Assessment

After the exudate volume assessment, 20 µL of exudate was removed to count the cell migration in the necrotic region. The exudate was diluted using PBS; 20 µL of exudate to 180 µL of PBS (1/10). Twenty microliters was collected and diluted using 180 µL of methylene blue (1/10). The total number of cells was quantified in a Neubauer chamber [67].

##### Necrotic Tissue Area Analysis

The area of necrosis (2 × 1 cm^2^) and the total area (4 × 2.5 cm^2^) were photographed using a digital camera Samsung (Samsung, Manaus, Brazil, 12.1 megapixels), and the ratio between these areas was presented as a percentage (%) after complete removal from the injured area. The images were analyzed using the Image J 1.3.1 program (National Institute of Mental Health, Bathesda, MD, USA), suitable for calculating areas [67]. 

##### Nitric Oxide Activity Evaluation 

The production of nitric oxide from the exudate supernatant was evaluated through the quantification of its metabolite nitrite, using the Griess Reagent method [68]. After 10 min of reaction, the samples were read at a wavelength of 540 nm. The nitrite concentrations in the samples were determined through the factor obtained from the standard curve, with serial dilutions of sodium nitrite conducted at known concentrations. To exclude interference from protein accumulation in the exudate during analysis by the ELISA reader, samples were centrifuged for 5 min at 3000 rpm before the conduction of the standard procedure [68].

#### 3.5.4. Histopathological Evaluation

##### Hematoxylin and Eosin Evaluation

The effect of the hydrogel dressing on the lesions, was evaluated using histology. The injured tissues were preserved in formaldehyde (10%) for 24 h, and then cryoprotected in sucrose (20%). The samples were embedded in paraffin, the sections were prepared using a microtome and stained with hematoxylin/eosin. The histological sample from the cryostat were soaked in alcohol (99%, 95% and 70%), washed with running water (5 min), smeared with hematoxylin (10 min) and washed again under running water (5 min). The tissue was then dappled with eosin (5 min), washed with running water (5 min) and dehydrated in 70%, 95%, 99% and absolute alcohol. The samples were then cleared in xylol I and II (5 min), after which they were mounted on slides for microscopic analysis [69,70].

##### Collagen Evaluation Using Picrosirius Red 

Histological sections of 40 µm were rinsed using running water, and the Picrosirius dye was placed on the tissues (30 min). The slides were washed under running water (3 min), and after drying they were covered with Hematoxylin Carazzi (1 min) and washed under running water again (5 min). After staining, the slides were analyzed under brightfield microscopy [67]. 

### 3.6. Statistical Analyses

The data was analyzed using mean, standard deviation and analysis of variance (ANOVA). F-test was used to compare the samples and the differences between means were detected by the Tukey using Graphpad Prism 7.0. A *p*-value less than 0.05 was considered statistically significant.

## 4. Conclusions

Polyacrylamide hydrogel synthesized in association with the calendula extract showed advantages in its use as a dressing for wound healing. The application of the hydrogel in the in vivo study promoted tissue regeneration and accelerated the healing process, positively regulating the expression of the main growth factors and reducing the production of pro-inflammatory factors. Results also showed an increase in the number of collagen fibers. The production of collagen fibers occurred through fibroblasts, the staining demonstrates the increase in birefringence of this material, qualitatively demonstrating an increase in newly formed collagen fibers and this, based on the healing cascade, is associated with intense fibroplasia in the tissue. No signs were seen of dermal or systemic toxicity. In addition, the polyacrylamide biomaterial bandages were easy to apply and remove from lesions after treatment. Although in some parameters of evaluation of the healing activity of HDSC and HDCC they were similar, it is believed that the study was promising. An increase in calendula concentration can heighten the efficiency of the wound healing process, as calendula is a species well known for its healing and anti-inflammatory potential. The positive results contribute to the technological development of the product, which is expected to be used in the future in the creation of a topical herbal therapy application for wound treatment.

## Data Availability

Data generated and analyzed this study are included in this article.
